# Small-pox—a Suggestion

**Published:** 1880-03

**Authors:** John Conant


### ARTICLE IX.

### SMALL-POX.—A SUGGESTION.

ROCKFORD, ILLINOIS, Feb. 12th, 1880.—MESSRS. EDITORS: I notice by the Chicago papers there are some cases of small-pox in the city. Just after the war I had charge of a small-pox hospital at Prairie du Chien, Wisconsin. I succeeded in many instances in preventing the pustules from filling, and aborting the disease, so that the patients did not show any marks or pits a few weeks after recovery. In one instance, a young lady, who would have had confluent small-pox, has not a mark or pit visible to-day upon the face. The remedy is cream of tartar and water; give a sufficient quantity to operate as laxative and diuretic, and continue the treatment as it becomes necessary. The local application, of course, should not be omitted. I should think vaseline or carbolated cosmoline would be a fine external application. This treatment for small-pox may not be new to you, but I am satisfied it is not in general use, and for that reason have mentioned it to you. I shall remain here several weeks, and will be pleased to hear from you. With kind regards, I remain very truly yours, JOHN CONANT.

